# A Temperature-Dependent Model of Shape Memory Alloys Considering Tensile-Compressive Asymmetry and the Ratcheting Effect

**DOI:** 10.3390/ma13143116

**Published:** 2020-07-13

**Authors:** Longfei Wang, Peihua Feng, Ying Wu, Zishun Liu

**Affiliations:** 1 State Key Laboratory for Strength and Vibration of Mechanical Structures, National Demonstration Center for Experimental Mechanics Education, Shaanxi Engineering Laboratory for Vibration Control of Aerospace Structures, School of Aerospace Engineering, Xi’an Jiaotong University, Xi’an 710049, China; wlfpal@stu.xjtu.edu.cn (L.W.); fphd2017@xjtu.edu.cn (P.F.); 2 International Center for Applied Mechanics, State Key Laboratory for Strength and Vibration of Mechanical Structures, School of Aerospace Engineering, Xi’an Jiaotong University, Xi’an 710049, China

**Keywords:** constitutive model, shape memory alloys, simulation, tensile-compressive asymmetry

## Abstract

Tensile-compressive asymmetry and the ratcheting effect are two significant characteristics of shape memory alloys (SMAs) during uniaxial cyclic tests, thus having received substantial attention in research. In this study, by redefining the internal variables in SMAs by considering the cyclic accumulation of residual martensite, we propose a constitutive model for SMAs to simultaneously reflect tensile-compressive asymmetry and the cyclic ratcheting effect under multiple cyclic tests. This constitutive model is temperature dependent and can be used to reasonably capture the typical features of SMAs during tensile-compressive cyclic tests, which include the pseudo-elasticity at higher temperatures as well as the shape-memory effect at lower temperatures. Moreover, the proposed model can predict the cyclic mechanical behavior of SMAs subjected to applied stresses with different peak and valley values under tension and compression. Agreement between the predictions obtained from the proposed model and the published experimental data is observed, which confirms that the proposed novel constitutive model of SMAs is feasible.

## 1. Introduction

Shape memory alloys (SMAs), as a new class of functional material [1,2,3,4] possessing many unique features (e.g., biocompatibility, pseudo-elasticity, and shape-memory effect), have been utilized in various fields over the last three decades, such as automotive engineering [5,6], the aerospace industry [7], robotics [8,9], medical implants [10,11], intelligent control engineering [12,13], structural reinforcement [14], and virtual reality technology [15]. In these applications, SMAs are often used as structural components and undergo repeated tensile-compressive cycles during service. The experimental results from some cyclic loading tests [16,17] show that SMAs demonstrate asymmetric behavior when they are subjected to tensile-compressive loadings, as well as the cyclic ratcheting deformation. Many attempts to capture these two significant features of SMAs have been made through proposing constitutive models to accurately describe the mechanical behavior of SMAs.

In describing the tensile-compressive asymmetry [18,19,20,21] of SMAs, Wasilewski [22] first reported the mechanical property variation and significant differences between the effects of tensile and compressive loading on SMAs. Subsequently, many constitutive models [23,24,25,26,27,28,29,30,31] exhibiting the tensile-compressive asymmetry of SMAs have been presented. For example, Paiva et al. [23] proposed a constitutive model considering both the tensile-compressive asymmetry and the plastic strains that occur in the thermomechanical processing of SMAs. Zaki et al. [27] extended their original Zaki-Moumni model to account for tensile-compressive asymmetry over a wide temperature range, and they developed the asymmetry model to analyze SMA cantilever beams subjected to tip loads [32]. Poorasadion et al. [28] further developed the original Brinson model [33] into a novel tensile-compressive asymmetry model for SMAs and successfully applied their model in a two-dimensional (2D) Euler–Bernoulli beam to predict its behavior through ABAQUS/Standard. Ravari et al. [30] later considered the tension-compression asymmetry and proposed a new three-dimensional (3D) constitutive law for SMAs based on the microplane theory. Recently, Wang et al. [31] redefined the martensite internal variable of SMAs based on the original Brinson model [33] and proposed a new constitutive model to predict the tensile-compressive asymmetry behavior of SMAs with better continuity and flexibility.

The ratcheting deformation, which is mainly caused by the increase in and, subsequent, accumulation of residual martensite during multiple cyclic tests [34,35], is another significant feature of SMAs and should be predicted in their applications. The ratcheting deformation of SMAs during cycling has been widely investigated experimentally by researchers in the last decade, including strain-controlled [36,37,38,39] and stress-controlled [17,40,41,42,43] cyclic loading tests. According to the experimental observations, several phenomenological constitutive models for SMAs have been developed to successfully predict ratcheting deformation during multiple cycles [44,45,46,47,48,49,50]. Tanaka et al. [44] introduced three internal variables into a macroscopic theoretical framework to interpret the hysteresis behavior of SMAs during thermomechanical cyclic loadings, and they also analyzed the subloops due to the incomplete transformations of the SMAs. Saint-Sulpice et al. [45] developed the constitutive equations of a novel 3D macroscopic model for SMAs which could reproduce all the experimental observations, in which a permanent inelastic strain occurs and increases during cyclic tests. On the basis of Kang’s experimental observations [17] for both pseudo-elastic and shape-memory SMAs, Kan and Kang [46] and Yu et al. [47] constructed the corresponding constitutive models to predict uniaxial transformation ratcheting deformation of pseudo-elastic and shape-memory SMAs, respectively. Xiao et al. [48] considered the effects of loading rate on the results of cyclic tests and presented a 3D thermomechanically coupled constitutive model to describe the rate-dependent cyclic performance of pseudo-elastic SMAs, in which the ratcheting deformation was included. Recently, Ashrafi [49] developed a constitutive model that considered the permanent strain evolution of SMAs under cyclic loading and was able to predict the hysteresis loop of SMAs and its changes along with the evolution of the permanent strain.

As mentioned above, the existing constitutive models for SMAs have already successfully described the features of tensile-compressive asymmetry or ratcheting deformation under multiple cyclic loadings. However, a constitutive model that can predict both tensile-compressive asymmetry and ratcheting deformation of the SMAs is rarely reported. In the model of Kan and Kang [46], they tried to take into account the ratcheting effect to predict the experimental observations of SMAs in uniaxial tensile-compressive cyclic tests [17]. Although their model reflected the tensile-compressive asymmetry and ratcheting deformation of SMAs during cycling, they considered only the mechanical features of SMAs at room temperature.

SMAs are a group of metallic alloys with thermal sensitivity, and therefore the models of SMAs should be temperature dependent [1]. Therefore, we redefine the internal variables in SMAs by considering the cyclic accumulation of residual martensite and propose a temperature-dependent constitutive model of SMAs that reflects the tensile-compressive asymmetry and ratcheting effect under different applied stresses. In Section 2, the temperature-dependent constitutive model for SMAs is presented. In Section 3, the corresponding simulated results based on the proposed model are obtained and discussed by comparing simulation results with the experimental ones. Finally, concluding remarks are drawn in Section 4.

## 2. Constitutive Model

The uniaxial transformation ratcheting of SMAs during tensile-compressive cycles is considered and the SMA constitutive law based on the Brinson model [33] can be obtained as:

(1) $$\sigma =D\left(\xi \right)\left(\varepsilon -\varepsilon_{in}^{\pm }\right)$$

where ξ and D(ξ) represent the martensite volume fraction and ξ-related elastic modulus, respectively (the martensite refers to the detwinned martensite in this study, which is distinguished from the twinned martensite). $\varepsilon_{in}^{\pm }$ is the total inelastic strain. The superscript ‘+’ represents the strain under tension, while the superscript ‘-’ represents the strain under compression. The total inelastic strain consists of two parts, i.e., the transformation strain $\varepsilon_{tr}^{\pm }$ and the cyclic ratcheting strain $\varepsilon_{p}^{\pm }$. Thus, Equation (1) can be further expressed as:

(2) $$\sigma =D\left(\xi \right)\left(\varepsilon -\varepsilon_{tr}^{\pm }-\varepsilon_{p}^{\pm }\right)$$

**Figure 1:**
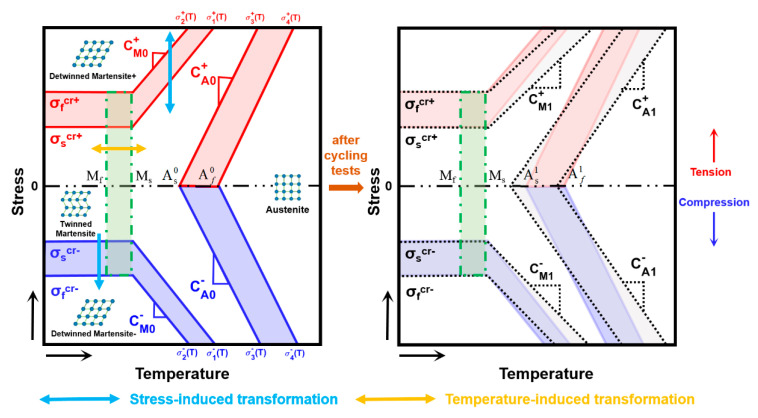
Asymmetric tensile-compressive phase diagram predicting the critical stresses for shape memory alloys (SMAs) before cycling tests (**left**) and after cycling tests (**right**). The solid lines represent the critical stresses for SMAs in the initial unstable state and the dotted lines represent the critical stresses for SMAs in the final stable state.

Since the elastic modulus D(ξ) of SMAs in Equation (2) is affected by ξ, the variation in ξ should be known. The temperature-stress phase diagram of SMAs, as shown in Figure 1, is always used to predict the variation of ξ given some material parameters and the critical stresses related to the evolution of ξ. As tension-compression asymmetry is considered, some material parameters discussed by Wang et al. [31] are defined in the left diagram in Figure 1, among them, $M_{s}$ and $M_{f}$ are the initial and final temperatures of the martensitic transformation, and $A_{s}^{0}$ and $A_{f}^{0}$ are the initial and final temperatures of inverse martensitic transformation. $\sigma_{s}^{cr+}$ and $\sigma_{f}^{cr+}$ represent the critical transformation stresses under tension when $T<M_{s}$, while $\sigma_{s}^{cr-}$ and $\sigma_{f}^{cr-}$ represent the critical transformation stresses under compression. $C_{M0}^{+}$ and $C_{A0}^{+}$ are the stress-temperature slopes, respectively, for the martensitic and inverse martensitic transformations under tension, and $C_{M0}^{-}$ and $C_{A0}^{-}$ are the corresponding slopes under compression. Moreover, the tensile parameter and the corresponding compressive parameter are most likely different, for example, $\sigma_{s}^{cr+}\neq \sigma_{s}^{cr-}$ and $C_{M0}^{+}\neq C_{M0}^{-}$ [31]. Based on these parameters, the critical transformation stresses under tension (red lines in the left diagram in Figure 1, which are marked by $\sigma_{1}^{+}\left(T\right)$, $\sigma_{2}^{+}\left(T\right)$, $\sigma_{3}^{+}\left(T\right)$ and $\sigma_{4}^{+}\left(T\right)$) and under compression (blue lines in the left diagram in Figure 1, which are marked by $\sigma_{1}^{-}\left(T\right)$, $\sigma_{2}^{-}\left(T\right)$, $\sigma_{3}^{-}\left(T\right)$ and $\sigma_{4}^{-}\left(T\right)$), as well as the phase transformation regions under tension (light red areas in the left diagram in Figure 1) and under compression (light blue areas in the left diagram in Figure 1) can be well determined. Additionally, the areas representing fully austenite, fully martensite (i.e., fully detwinned martensite), and fully twinned martensite are divided by these critical stresses (see the left diagram in Figure 1). The variation in ξ, which is affected by temperature and stress, can be reflected during phase transformation according to Figure 1.

With the consideration of the ratcheting effect, some material parameters of SMAs in the left diagram in Figure 1 can change after cyclic tests, as shown in the right diagram in Figure 1. Therefore, more material parameters for stable cycles should be defined. According to some studies [17,51,52], the material parameters $\sigma_{s}^{cr\pm }$, $\sigma_{f}^{cr\pm }$, $M_{s}$, and $M_{f}$ change minimally when the state of the SMAs changes from unstable to stable (i.e., ratcheting deformation hardly occurs when $T<M_{s}$) after cyclic tests, and therefore we only redefine $C_{A0}^{\pm }$, $C_{M0}^{\pm }$, $A_{s}^{0}$ and $A_{f}^{0}$ as the corresponding initial unstable material parameters (left diagram in Figure 1) and regard $C_{A1}^{\pm }$, $C_{M1}^{\pm }$, $A_{s}^{1}$ and $A_{f}^{1}$ as the corresponding stable parameters after certain cycles (right diagram in Figure 1). Critical stresses of SMAs after the cyclic tests (black dotted lines in the right diagram in Figure 1) can be obtained by these stable parameters. In addition, two green dash-dotted lines in Figure 1 are controlled by $M_{s}$ and $M_{f}$ and represent the critical temperatures, between which the phase transformation of austenite-twinned martensite occurs. With the consideration of all these parameters shown in Figure 1, the critical stresses of SMAs during evolution can be obtained and the variation in ξ that takes into account the tensile-compressive asymmetry and the ratcheting effect under multiple loading cycles can be effectively predicted.

Due to the ratcheting effect, the martensite partially remains in the SMAs after each unloading process and accumulates for multiple loading cycles. Therefore, the martensite volume fraction ξ in Equation (2) can be divided into the following two parts: $\xi_{ir}$ which is irreversible and $\xi_{r}$ which is reversible.

The irreversible martensite volume fraction $\xi_{ir}$ can be obtained as follows:

(3) $$\xi_{ir}=\left[\xi_{ir\_\max}^{+}k_{0}^{+}\left(\sigma_{p}^{+}\right)+\xi_{ir\_\max}^{-}k_{0}^{-}\left(\sigma_{p}^{-}\right)\right]\left(1-e^{-\psi_{ir}\xi_{c}}\right)$$

where $\xi_{ir\_\max}^{+}$ and $\xi_{ir\_\max}^{-}$ represent the maximum irreversible martensite volume fraction under tension and compression, respectively, $\sigma_{p}^{\pm }$ is the peak of the applied tensile stress or compressive stress and $\psi_{ir}$ is the controlling parameter. $\xi_{c}$ is a ξ-related function representing the accumulation of martensite volume fraction and can be expressed as $\xi_{c}=\int_{0}^{t}\left|\dot{\xi }\left(\tau \right)\right|d\tau$ [46]. $k_{0}^{\pm }\left(\sigma_{p}^{\pm }\right)$ in Equation (3) is used to capture the maximum values of $\xi_{ir}$ under tension and compression by considering different values of $\sigma_{p}^{\pm }$. It can be developed on the basis of a previous study [31] by using a compound function that describes the variation of variant volume fraction in SMAs under tension or compression as:

(4) $$k_{0}^{\pm }\left(\sigma_{p}^{\pm }\right)=\left(1/\pi \right)\arctan\left\{2\sinh\left[\kappa^{\pm }\left(\sigma_{p}^{\pm }-\left(\sigma_{10}^{\pm }\left(T\right)+\sigma_{20}^{\pm }\left(T\right)\right)/2\right)\right]\right\}+1/2$$

where $\sigma_{10}^{\pm }\left(T\right)$ and $\sigma_{20}^{\pm }\left(T\right)$ are two initial values of temperature-related critical transformation stresses, i.e., $\sigma_{1}^{\pm }\left(T\right)$ and $\sigma_{2}^{\pm }\left(T\right)$ in Figure 1, for the first tensile or compressive loading process. Since $\left|\sigma_{1}^{\pm }\left(T\right)\right|\leq \left|\sigma_{2}^{\pm }\left(T\right)\right|$ according to Figure 1, it can be obtained that $\left|\sigma_{10}^{\pm }\left(T\right)\right|\leq \left|\sigma_{20}^{\pm }\left(T\right)\right|$. $\kappa^{\pm }$ in Equation (4) is the pseudo-hardening coefficient for tension or compression that can be used to measure the evolution rate of the variants in SMAs. Note that Equation (4) is a special case as it can only predict the variant evolution for the first loading cycle. Generally, $\sigma_{1}^{\pm }\left(T\right)$ and $\sigma_{2}^{\pm }\left(T\right)$ are varying for multiple cycles due to ratcheting effect and Equation (4) can be developed as:

(5) $$k^{\pm }\left(\sigma_{p}^{\pm }\right)=\left(1/\pi \right)\arctan\left\{2\sinh\left[\kappa^{\pm }\left(\sigma_{p}^{\pm }-\left(\sigma_{1}^{\pm }\left(T\right)+\sigma_{2}^{\pm }\left(T\right)\right)/2\right)\right]\right\}+1/2$$

Note that we take advantage of continuity of the inverse trigonometric function and divergence of the hyperbolic sine function in the compound function of Equation (5) to describe the variant evolution of SMAs. Therefore, Equation (5) is better than some other evolution functions in continuity and smoothness [31]. The use of κ in Equation (5) also makes the evolution rate of variants in SMAs controllable, which leads to Equation (5) being more flexible to use. We set an error function to monitor the tendency of the function value towards 0 or 1 when the critical stress is applied, and the error function is written as:

(6) $$\delta \left(\kappa^{\pm }\right)=\left\{1/2-\left(1/\pi \right)\arctan\left[2\sinh\left(\kappa^{\pm }\tilde{\sigma }/2\right)\right]\right\}\times 100\%$$

where $\tilde{\sigma }$ is the difference in value between the critical stresses for the pseudo-hardening processes. It should be noted that when $\delta \left(\kappa^{\pm }\right)$ decreases, the evolution rate of the variant volume fraction curve becomes higher.

On the basis of the Brinson model [33], we redefine the reversible martensite volume fraction $\xi_{r}$ by using the functional form of Equation (5) and considering the effect of $\xi_{ir}$. It can be expressed as:

(7) $$\begin{array}{l} \xi_{r}=\xi_{r}|{}_{x,y}^{\pm }\\ =\left\{\left\{\left(1/\pi \right)\arctan\left\{2\sinh\left[\kappa^{\pm }\left(\sigma -\left(\sigma_{x}^{\pm }(T)+\sigma_{y}^{\pm }(T)\right)/2\right)\right]\right\}-1/2\right\}{\left(1-\Psi \right)}^{j-1}+1\right\}\left[1-\left(\xi_{ir\_\max}^{+}k_{0}^{+}+\xi_{ir\_\max}^{-}k_{0}^{-}\right)\left(1-e^{-\psi_{ir}\xi_{c}}\right)\right] \end{array}$$

where $\sigma_{x}^{\pm }\left(T\right)$ and $\sigma_{y}^{\pm }\left(T\right)$ are the phase transformation critical stresses for tension or compression of the SMAs. x=1 and y=2 are assigned to the loading process, whereas x=3 and y=4 are assigned to the unloading process, respectively. The relations of $\left|\sigma_{1}^{\pm }\left(T\right)\right|\leq \left|\sigma_{2}^{\pm }\left(T\right)\right|$ and $\left|\sigma_{4}^{\pm }\left(T\right)\right|\leq \left|\sigma_{3}^{\pm }\left(T\right)\right|$ are recognized clearly according to Figure 1. j is the number of the tension-unloading and compression-unloading processes, for example, j=1 during the first tension-unloading process, while j=2 when the compression-unloading process first occurs, etc. Ψ in Equation (7) is a constant value related to the first tensile unloading or compressive unloading, and can be obtained by:$\Psi =\left(1/\pi \right)\arctan\left\{2\sinh\left[\kappa^{\pm }\left(-\left(\sigma_{30}^{\pm }\left(T\right)+\sigma_{40}^{\pm }\left(T\right)\right)/2\right)\right]\right\}+1/2$, among which $\sigma_{30}^{\pm }\left(T\right)$ and $\sigma_{40}^{\pm }\left(T\right)$ are initial value of critical stresses of $\sigma_{3}^{\pm }\left(T\right)$ and $\sigma_{4}^{\pm }\left(T\right)$ for the first tensile or compressive unloading process. Note that the physical significance of Ψ is the residual martensite volume fraction after the first tensile unloading (the superscript ”+” is assigned when the cycle begins with tension) or first compressive unloading (the superscript “–“ is assigned when the cycle begins with compression). The term ${\left(1-\Psi \right)}^{j-1}$ in Equation (7) is used to unify the description of $\xi_{r}$ to reflect the different features (e.g., the pseudo-elasticity at higher temperatures and the shape-memory effect at low temperatures) of SMAs at various temperatures. For multiple cycles, Ψ tends towards 0 and ${\left(1-\Psi \right)}^{j-1}$ approximately equal to 1 when SMAs show pseudo-elasticity at higher temperatures; therefore, the value of $\xi_{r}$ shows fluctuations between 0 and 1 with the effect of $\xi_{ir}$, representing the occurrence of the martensite phase transformation. When SMAs show shape-memory effects at lower temperatures, Ψ tends towards 1 and ${\left(1-\Psi \right)}^{j-1}$ is almost equal to 0 during cycling. As a result, $\xi_{r}$ is only related to $\xi_{ir}$ according to Equation (7), which means that the phase transformation rarely happens for shape-memory SMAs. Particularly, when SMAs show features between pseudo-elasticity and shape-memory effect under the temperature of $T\in \left(A_{s}^{0},A_{f}^{0}\right)$, Ψ∈(0,1) and ${\left(1-\Psi \right)}^{j-1}$ in Equation (7) is, then, regarded as an exponential zoom factor that is related to the number of cycle. With the effects of the zoom factor ${\left(1-\Psi \right)}^{j-1}$, the value of $\xi_{r}$ fluctuates with its valley value increasing during cycling, which implies the accumulation of residual martensite at various temperatures. The item ${\left(1-\Psi \right)}^{j-1}$ is used to provide a continuous transition of features of SMAs between pseudo-elasticity and shape-memory effect.

It should also be noted that Equation (7) has its limitations when the incomplete forward and inverse martensite transformations are considered. Thus, we set ${\xi_{r}^{0}|}_{1,2}^{\pm }\left(\sigma_{p}^{\pm }\right)$ as $\sigma_{p}^{\pm }$-related initial value of $\xi_{r}$ when each tensile or compressive unloading process happens and rewrite Equation (7) as:

(8) $$\xi_{r}=\{\begin{array}{ll} \xi_{r}|{}_{1,2}^{\pm } & \left(loading\right)\\ \xi_{r}^{0}|{}_{1,2}^{\pm }\left(\sigma_{p}^{\pm }\right)\xi_{r}|{}_{3,4}^{\pm } & \left(unloading\right) \end{array}$$

For simplification, we set $P_{x,y}^{\pm }$ as the stress induced martensite volume fraction without considering ratcheting deformation during cycling and express it in a simple form as:

(9) $$P_{x,y}^{\pm }=\left(1/\pi \right)\arctan\left\{2\sinh\left[\kappa^{\pm }\left(\sigma -\left(\sigma_{x}^{\pm }\left(T\right)+\sigma_{y}^{\pm }\left(T\right)\right)/2\right)\right]\right\}+1/2$$

In addition, we set

(10) $${\bar{P}}_{x,y}^{\pm }=\left(1/\pi \right)\arctan\left\{2\sinh\left[\kappa^{\pm }\left(\sigma -\left(\sigma_{x}^{\pm }\left(T\right)+\sigma_{y}^{\pm }\left(T\right)\right)/2\right)\right]\right\}-1/2$$

Combining Equation (3) with Equation (8), the total martensite volume fraction ξ can be expressed as:

(11) $$\xi =\xi_{r}+\xi_{ir}=\{\begin{array}{l} \begin{array}{ll} {\bar{P}}_{1,2}^{\pm }\left[1-\left(\xi_{ir\_\max}^{+}k_{0}^{+}+\xi_{ir\_\max}^{-}k_{0}^{-}\right)\left(1-e^{-\psi_{ir}\xi_{c}}\right)\right]{\left(1-\Psi \right)}^{j-1}+1 & \left(loading\right) \end{array}\\ \begin{array}{ll} {\xi_{r}^{0}|}_{1,2}^{\pm }\left(\sigma_{p}^{\pm }\right){\bar{P}}_{3,4}^{\pm }\left[1-\left(\xi_{ir\_\max}^{+}k_{0}^{+}+\xi_{ir\_\max}^{-}k_{0}^{-}\right)\left(1-e^{-\psi_{ir}\xi_{c}}\right)\right]{\left(1-\Psi \right)}^{j-1}\\ +{\xi_{r}^{0}|}_{1,2}^{\pm }\left(\sigma_{p}^{\pm }\right)+\left[1-{\xi_{r}^{0}|}_{1,2}^{\pm }\left(\sigma_{p}^{\pm }\right)\right]\left(\xi_{ir\_\max}^{+}k_{0}^{+}+\xi_{ir\_\max}^{-}k_{0}^{-}\right)\left(1-e^{-\psi_{ir}\xi_{c}}\right) & \left(unloading\right) \end{array} \end{array}$$

According to Equation (11), the variation in ξ as well as the ξ-related elastic modulus D(ξ) in Equation (2) can be obtained.

The transformation strain $\varepsilon_{tr}^{\pm }$ in Equation (2), as a component of the total strain ε, is induced by phase transformation during the tensile-compressive cycles and expressed as:

(12) $${\varepsilon_{tr}|}_{x,y}^{\pm }=\varepsilon_{L}^{\pm }{{\bar{\xi }}_{r}|}_{x,y}^{\pm }$$

where $\varepsilon_{L}^{\pm }$ represents the maximum transformation strain under tension or compression. ${{\bar{\xi }}_{r}|}_{x,y}^{\pm }=P_{x,y}^{\pm }$ represents the reversible martensite volume fraction at various temperatures without considering the ratcheting deformation. Setting $P_{1,2}^{0\pm }\left(\sigma_{p}^{\pm }\right)$ as the initial value of $P_{x,y}^{\pm }$ when each tensile or compressive unloading happens and considering the incomplete transformations when $\sigma_{p}^{+}<\sigma_{2}^{+}\left(T\right)$ or $\sigma_{p}^{-}<\sigma_{2}^{-}\left(T\right)$, we can further express ${{\bar{\xi }}_{r}|}_{x,y}^{\pm }$ as:

(13) $${{\bar{\xi }}_{r}|}_{x,y}^{\pm }=\{\begin{array}{ll} P_{1,2}^{\pm } & \left(loading\right)\\ P_{1,2}^{0\pm }\left(\sigma_{p}^{\pm }\right)P_{3,4}^{\pm } & \left(unloading\right) \end{array}$$

The cyclic ratcheting strain $\varepsilon_{p}^{\pm }$ in Equation (2), which is quite different from $\varepsilon_{tr}^{\pm }$, is a type of accumulated strain and can be expressed by using $\xi_{c}$ as:

(14) $$\varepsilon_{p}^{\pm }=\xi_{ir}\varepsilon_{M}^{\pm }=\pm \left(\varepsilon_{sa}^{+}k_{0}^{+}+\varepsilon_{sa}^{-}k_{0}^{-}\right)\left(1-e^{-\psi_{2}\xi_{c}}\right)$$

and

(15) $$\varepsilon_{M}^{\pm }=\pm \left(\varepsilon_{sa}^{+}k_{0}^{+}+\varepsilon_{sa}^{-}k_{0}^{-}\right)\left(1-e^{-\psi_{2}\xi_{c}}\right)/\left[\left(\xi_{ir\_\max}^{+}k_{0}^{+}+\xi_{ir\_\max}^{-}k_{0}^{-}\right)\left(1-e^{-\psi_{ir}\xi_{c}}\right)\right]$$

where $\varepsilon_{sa}^{\pm }$ represents the saturation value of the ratcheting strain induced by tension or compression, while $\psi_{2}$ is the controlling parameter for the accumulation rate of $\varepsilon_{p}^{\pm }$.

It should be highlighted that the critical transformation stresses shown in Figure 1, i.e., $\sigma_{1}^{\pm }\left(T\right)$, $\sigma_{2}^{\pm }\left(T\right)$, $\sigma_{3}^{\pm }\left(T\right)$, and $\sigma_{4}^{\pm }\left(T\right)$, change progressively from their initial values to stable ones (see Figure 1). Therefore, the evolution rule of these critical stresses during cycling should be given as:

(16) $$\sigma_{X}^{\pm }=\sigma_{X0}^{\pm }-\left(\sigma_{X0}^{\pm }-\sigma_{X1}^{\pm }\right)\left(1-e^{-\gamma_{X}^{\pm }\xi_{c}}\right)$$

where *X* = 1, 2, 3, 4. $\sigma_{X0}^{\pm }$ represents the initial value of each critical stress for the first tension-unloading or compression-unloading process, while $\sigma_{X1}^{\pm }$ represents stable value of each critical stress after evolution. These initial and steady critical stresses are calculated according to the material parameters in Figure 1. $\gamma_{X}^{\pm }$ in Equation (16) is the corresponding controlling parameter used to reflect the saturation rate of each critical stress during evolution.

Moreover, it is noted that the maximum transformation strain $\varepsilon_{L}^{\pm }$ in Equation (12) changes after evolution, since the forward martensitic transformation is hindered by the dislocation slip in SMAs due to the cyclic ratcheting effect [17,45]. The evolution rate of the pseudo-hardening process always changes to a higher value according to experimental findings [17]. Therefore, evolution equations of $\varepsilon_{L}^{\pm }$ and $\kappa^{\pm }$ which are related to the pseudo-hardening stage, are given by inducing $\xi_{c}$:

(17) $$\varepsilon_{L}^{\pm }=\varepsilon_{L0}^{\pm }-\left(\varepsilon_{L0}^{\pm }-\varepsilon_{L1}^{\pm }\right)\left(1-e^{-\gamma_{5}^{\pm }\xi_{c}}\right)$$

(18) $$\kappa^{\pm }=\kappa_{0}^{\pm }-\left(\kappa_{0}^{\pm }-\kappa_{1}^{\pm }\right)\left(1-e^{-\gamma_{6}^{\pm }\xi_{c}}\right)$$

where $\varepsilon_{L0}^{\pm }$ and $\varepsilon_{L1}^{\pm }$ are the maximum transformation strains for the first and steady tensile-compressive cycles, respectively. Similarly, $\kappa_{0}^{\pm }$ and $\kappa_{1}^{\pm }$ represent the pseudo-hardening coefficients for the first and steady cycles, respectively. $\gamma_{5}^{\pm }$ and $\gamma_{6}^{\pm }$ are the controlling parameters governing the saturation rates corresponding to $\varepsilon_{L}^{\pm }$ and $\kappa^{\pm }$ during cycling.

The temperature-induced phase transformation between austenite and twinned martensite (green region in Figure 1) can be predicted by setting

(19) $$R=\left(1/\pi \right)\arctan\left\{2\sinh\left[\kappa^{R}\left(T-\left(M_{s}+M_{f}\right)/2\right)\right]\right\}+1/2$$

where $\kappa^{R}$ is the pseudo-hardening coefficient managing the evolution rate of the temperature-induced phase transformation.

Substituting Equations (3)–(19) into Equation (2), we finally obtain the temperature-dependent model of SMAs reflecting the ratcheting deformation during tensile-compressive cycles as:

(20) $$\sigma =D_{x,y}^{\pm }\left(\xi_{x,y}^{\pm }\right)\left[\varepsilon -\left(\varepsilon_{L}^{\pm }-\varepsilon_{e}\right){{\bar{\xi }}_{r}|}_{x,y}^{\pm }-\varepsilon_{M}^{\pm }\xi_{ir}-\varepsilon_{e}-\vartheta \right]$$

Note that $D_{x,y}^{\pm }\left(\xi_{x,y}^{\pm }\right)$ in Equation (20) is developed by considering four stages (including tensile loading, tensile unloading, compressive loading, and compressive unloading) of a complete tensile-compressive cycle on the basis of D(ξ) in Equation (2) and can be further expressed as $D_{x,y}^{\pm }\left(\xi_{x,y}^{\pm }\right)=\left[\left(D_{A}-D_{M}^{T}\right)R+D_{M}^{T}\right]\left(1-\xi_{x,y}^{\pm }\right)+D_{M}^{DT(\pm )}\xi_{x,y}^{\pm }$. Among them, $D_{A}$, $D_{M}^{DT\left(\pm \right)}$ and $D_{M}^{T}$ are the elastic moduli of SMAs in the fully austenite, the fully detwinned martensite (under tension or compression) and the fully twinned martensite, respectively.

In addition, there are still two correction functions, $\varepsilon_{e}$ and ϑ, in Equation (20) that should be highlighted. $\varepsilon_{e}$ is an accumulated function that corrects the strain error caused by the asymptotic functions of Equation (9) and Equation (10) [31] and makes the constitutive relation of SMAs continuous and smooth. As the correction function $\varepsilon_{e}$ is a type of accumulated function, we show the deduction and general form of its expression in Table 1. ϑ is the other correction function used to maintain continuity of the constitutive model when the tensile-compressive conversion occurs (i.e., the tensile unloading ends and compressive loading begins or vice versa). The correction function of ϑ is depended on $\varepsilon_{e}$, whose deduction and general form are also listed in Table 1.

## 3. Model Predictions and Discussions

**Figure 2:**
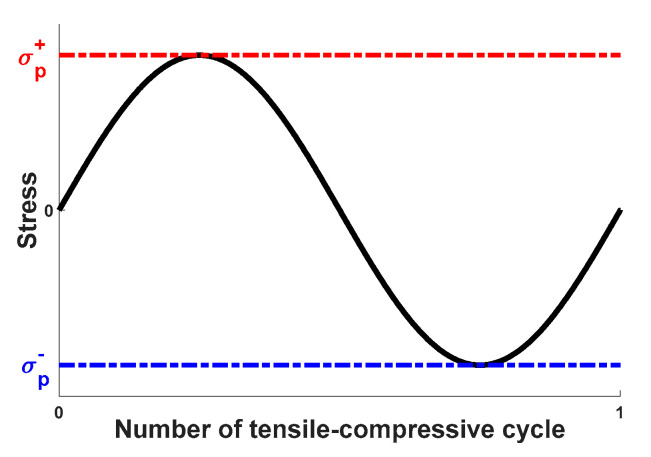
Uniaxial tensile-compressive loading charts with peak stresses of $\sigma_{p}^{+}$ under tension and $\sigma_{p}^{-}$ under compression.

In this section, we perform numerical tests to illustrate the capability of the proposed model described in Equation (20) for predicting the thermomechanical behavior of SMAs involving the tensile-compressive asymmetry and cyclic ratcheting deformation. Unless noted otherwise, a uniaxial tensile-compressive loading path, which is in the form of a sine wave, is employed and repeated for multiple cycles in this study, as shown in Figure 2. 

$\sigma_{p}^{+}$ and $\sigma_{p}^{-}$ in Figure 2 represent the peak values of applied stresses under tension and compression, respectively. In the following studies, we first fix the temperature and evaluate the predictions of the proposed constitutive model for pseudo-elastic SMAs and shape-memory SMAs, respectively. Then, we use the proposed model to predict the temperature-dependent behavior of SMAs, which can make this novel model more general and flexible to use. Predictions of the proposed model are compared with experimental data [17] available in this section.

## 3.1. Model Predictions of Pseudo-Elastic SMAs at a Fixed Temperature

In this section, we focus on model predictions for types of SMAs that show pseudo-elasticity at a fixed temperature. According to the study of Kan and Kang [46], the corresponding material parameters of Equation (20) used for prediction of the pseudo-elastic SMAs at room temperature are listed below in Table 2 (the critical stresses of SMAs at the fixed temperatures are given in Table 2).

The value of $D_{M}^{T}$ is not given in Table 2, since the twinned martensite does not exist in pseudo-elastic SMAs during stress-induced phase transformation. According to Equation (6), the error function for each corresponding coefficient κ is known and can be listed as $\delta \left(\kappa_{0}^{+}\right)=0.05\%/0.042\%$, $\delta \left(\kappa_{1}^{+}\right)=3.74\%/3.77\%$, $\delta \left(\kappa_{0}^{-}\right)=3.89\%/4.96\%$ and $\delta \left(\kappa_{1}^{-}\right)=8.1\%/11.96\%$. Two values for each κ represent the error values for the loading and unloading processes. It should be noted that the error values both under tension and compression become greater as the cycle becomes steady, which means that the evolution rate of the pseudo-hardening process becomes lower and the pseudo-hardening stress range becomes greater after certain cycles.

**Figure 3:**
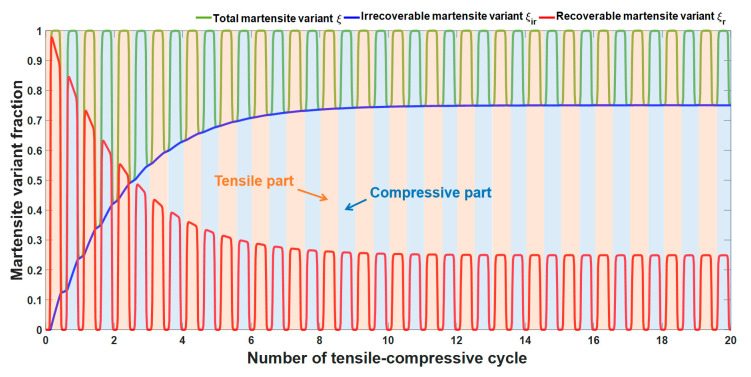
Variations in the martensite variants in SMAs during tensile-compressive cycles (the cycles start with a tensile process).

To understand the micro-mechanism of the ratcheting deformations of SMAs under tensile-compressive cycles, we first use the material parameters listed in Table 2 and predict the variations of inner variants in SMAs based on Equation (3), Equation (8), and Equation (11). Assuming that $\sigma_{p}^{+}\geq \sigma_{2}^{+}\left(T\right)$ and $\sigma_{p}^{-}\leq \sigma_{2}^{-}\left(T\right)$, we obtain the complete martensitic transformation under both tension and compression and plot the responses of martensite variant fractions in SMAs, involving $\xi_{ir}$, $\xi_{r}$ and ξ, in Figure 3. Note that the light red areas and the light blue areas in Figure 3 representing the martensitic transformation of SMAs occurs in the tension-unloading and compression-unloading processes, respectively. The irrecoverable martensite variant fraction $\xi_{ir}$, the variation in which is shown in Figure 3, progressively increases during the tensile-compressive cycles and finally approaches 0.751 when the cycle is steady. The recoverable martensite variant fraction, $\xi_{r}$, alternates and sustains fluctuations among the tensile and compressive regions. The reason is that the martensite in SMAs is formed from the original austenite either by tensile loading or by compressive loading and convert back to its original phase via the unloading process. However, the peak value of $\xi_{r}$ for each tensile-compressive cycle decreases and tends towards 0.249 after certain cycles under the influence of $\xi_{ir}$. The total martensite ξ, as the sum of $\xi_{r}$ and $\xi_{ir}$, fluctuates as the valley values increase under both tension and compression. The simulated results in Figure 3 illustrate the reason for the ratcheting deformation of SMAs, i.e., the incomplete inverse martensite phase transformation and the accumulation of irrecoverable martensite under tension and compression. In addition, the elastic modulus D(ξ) in Equation (20) depends on ξ; therefore, the variation in ξ plays an important role in the macroscopic description of the mechanical behavior for SMAs.

**Figure 4:**
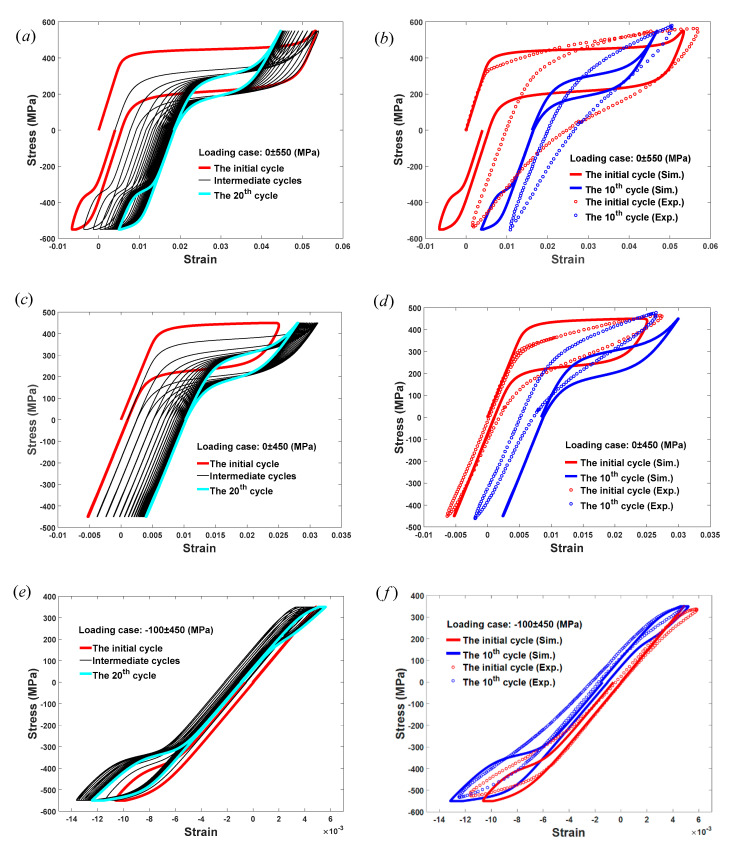
Model predictions and experimental verifications of the proposed constitutive model used for pseudo-elastic SMAs. (**a**) Model prediction under loading with stress range of 0±550 MPa; (**b**) Experimental verification under loading with stress range of 0±550 MPa; (**c**) Model prediction under loading with stress range of 0±450 MPa; (**d**) Experimental verification under loading with stress range of 0±450 MPa; (**e**) Model prediction under loading with stress range of −100 ± 450 MPa; (**f**) Experimental verification under loading with stress range of −100 ± 450 MPa. (“Sim.” represents the simulation results and “Exp.” represents the experimental results).

With the description of ξ-related D(ξ), the strain–stress relationships of the pseudo-elastic SMAs under tensile-compressive cyclic loadings with different peak stress values are plotted in Figure 4. It can be seen from Figure 4a,c,e that the proposed model is able to predict the ratcheting deformation of pseudo-elastic SMAs occurring in the uniaxial cyclic tensile-compressive cycles for loading cases with stress ranges of 0 ± 550 MPa, 0±450 MPa, and −100±450 MPa. Regarding Figure 4a, it can be clearly observed that when the applied stress is 0±550 MPa, the first tensile-compressive cycle of SMAs is formed with greater absolute values of critical stresses, greater transformation strain and ratcheting deformation, and a smaller stress range of pseudo-hardening. With the increase in loading cycles, for example, the 20th cycle, it is recognized that the initial cycle is replaced by a smaller cycle, which means that the absolute values of critical stresses, transformation strain, and ratcheting deformation all decrease, while the stress range of pseudo-hardening changes to a higher value. As a result, the area of the hysteresis loop of pseudo-elastic SMAs decreases during cycling, leading to a smaller dissipated energy of SMAs. In addition, it should be noted that the absolute values of compressive critical stresses are always larger than those under tension [53], and these initial critical stresses are not all reached in the simulated test by applying a loading whose range is 0±550 MPa. When the range of the applied stress changes from 0±550 MPa to a smaller one, namely 0±450 MPa as shown in Figure 4c, much more austenite remains in the SMAs in the loading process due to the incomplete forward martensitic transformation under this stress level, which leads to both the transformation strain and ratcheting deformation of SMAs becoming smaller for initial and steady cycles as compared with the results shown in Figure 4a. The hysteresis loops under compression are almost invisible in Figure 4c because the peak value of the applied compressive stress is smaller than $\sigma_{30}^{-}$. Moreover, the capability of dissipating energy for pseudo-elastic SMAs under a loading range of 0±450 MPa is weaker than that under a loading range of 0±550 MPa, since smaller hysteresis loop areas can be observed during cycling in Figure 4c. When the range of applied stress changes to −100±450 MPa, as shown in Figure 4e, a series of small hysteresis loops with very small transformation strain and ratcheting deformation are clearly shown under tension, since the peak tensile stress is far less than the critical stress $\sigma_{20}^{+}$ and $\sigma_{21}^{+}$. However, obvious loops can be seen under compression, which is similar to the results in Figure 4a. The results shown in Figure 4a,c,e effectively predict the strain–stress relations of SMAs under different stress ranges. Moreover, by using the evolution function of Equation (9) and Equation (10), the curves in these results are more flexible to use for prediction with better continuity and smoothness than those of other models [46].

Three sets of experimental observations involving tests with applied stress ranges of 0±550 MPa, 0±450 MPa, and −100±450 MPa, as reported by Kang et al. [17], are induced to assess these predictions, as shown in Figure 4b,d,f. Good agreement between the model predictions and experimental data can be obtained. These comparisons between model predictions and experimental observations all confirm the reliability of the proposed model for the prediction of the mechanical behavior of pseudo-elastic SMAs.

### 3.2. Model Predictions of Shape-Memory SMAs at a Fixed Temperature

Unlike pseudo-elastic SMAs, shape-memory SMAs are types of SMAs with strain remaining after unloading, and the residual strain can be recovered by subsequent heat treatment. In this section, we focus on the model predictions of shape-memory SMAs during tensile-compressive cycling. The corresponding material parameters of Equation (20) used for prediction of the shape-memory SMAs at room temperature are listed in Table 3 [46].

Associating the values of κ listed in Table 3 with Equation (6), we can obtain error values for each corresponding coefficient κ of shape-memory SMAs, namely, $\delta \left(\kappa_{0}^{+}\right)=0.08\%$, $\delta \left(\kappa_{1}^{+}\right)=7.34\%$, $\delta \left(\kappa_{0}^{-}\right)=0.18\%$ and $\delta \left(\kappa_{1}^{-}\right)=9.18\%$. Note that the critical stress for tensile or compressive unloading is not considered for shape-memory SMAs, and linear unloading is assumed; therefore, only one value is assigned for each δ(κ). Moreover, the evolution rate of the pseudo-hardening process becomes lower and the pseudo-hardening stress range becomes greater with increasing cycle number, since the error values increase.

**Figure 5:**
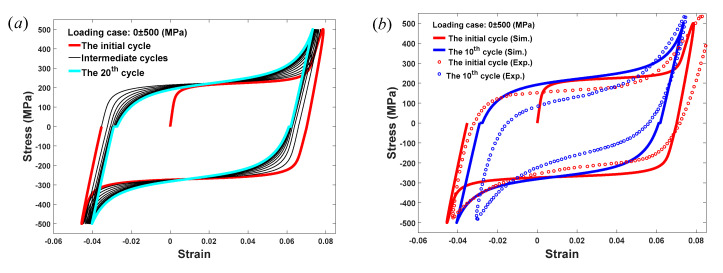

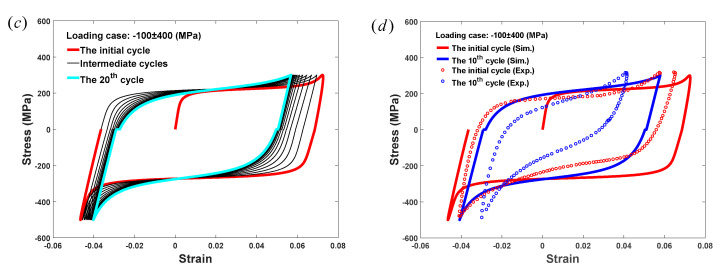
Model predictions and experimental verifications of the proposed constitutive model used for shape-memory SMAs. (**a**) Model prediction under loading with stress range of 0 ± 500 MPa; (**b**) Experimental verification under loading with stress range of 0 ± 500 MPa; (**c**) Model prediction under loading with stress range of −100 ± 400 MPa; and (**d**) Experimental verification under loading with stress range of −100 ± 400 MPa. (“Sim.” represents the simulation results and “Exp.” represents the experimental results).

According to these material parameters in Table 3 and Equation (20), the strain–stress relationships for shape-memory SMAs at room temperature under loadings with stress ranges of 0±500 MPa and −100±400 MPa are plotted in Figure 5. Note that the simulated results are similar in Figure 5a,c by applying different loadings, and these common features observed in Figure 5a,c can be summarized as follows: the residual strain, including a slight ratcheting strain [17] and the major strain caused by the rearrangement of martensite variants, remains after each tensile or compressive unloading, and the residual strain decreases during cycling; the transformation critical stresses remain the same during cycling, though the pseudo-hardening stress range becomes greater; and the dissipated energy of shape-memory SMAs for each cycle decreases as the loop area decreases. To verify the feasibility of the proposed model, we also show the corresponding comparisons between model predictions and experimental findings [17] in Figure 5b,d for different loading cases. The results prove that the obvious shape-memory effect of SMAs observed from these experimental findings for different loading cases can be reasonably captured by the proposed model.

### 3.3. Temperature-Dependent Model Predictions of SMAs at Various Temperatures

Since SMAs are alloys possessing both pseudo-elasticity and shape-memory effect at various temperatures and these two characteristics can be reciprocally transformed by varying the temperature, we use the proposed temperature-dependent constitutive model to predict the thermomechanical behavior of SMAs within a wide temperature range in this section.

In this section, the temperature-dependent critical stresses and their evolutions shown in Figure 1 should be calculated by using the material parameters. Therefore, we consider the Brinson’s model parameters [33] (these parameters can be experimentally determined [54]) and reasonably assume several material parameters in Figure 1 as a simulated example according to some studies [17,46,51,52,55,56]. The material parameters used in the proposed model for SMAs at various temperatures are listed below in Table 4.

Similarly, we list the following error values of the parameter κ: $\delta \left(\kappa_{0}^{+}\right)=5.64\%/0.21\%$, $\delta \left(\kappa_{1}^{+}\right)=10.57\%/2.47\%$, $\delta \left(\kappa_{0}^{-}\right)=5.64\%/0.28\%$ and $\delta \left(\kappa_{1}^{-}\right)=10.57\%/2.13\%$. These error values show the increasing tendency of the pseudo-hardening stress range for SMAs during cycling. In addition, the temperature-related coefficient $\kappa^{R}$, the error value of which is $\delta \left(\kappa^{R}\right)=0.29\%$, should be noted, as it reflects the conversion rate of the temperature-induced transformation between austenite and twinned martensite. 

**Figure 6:**
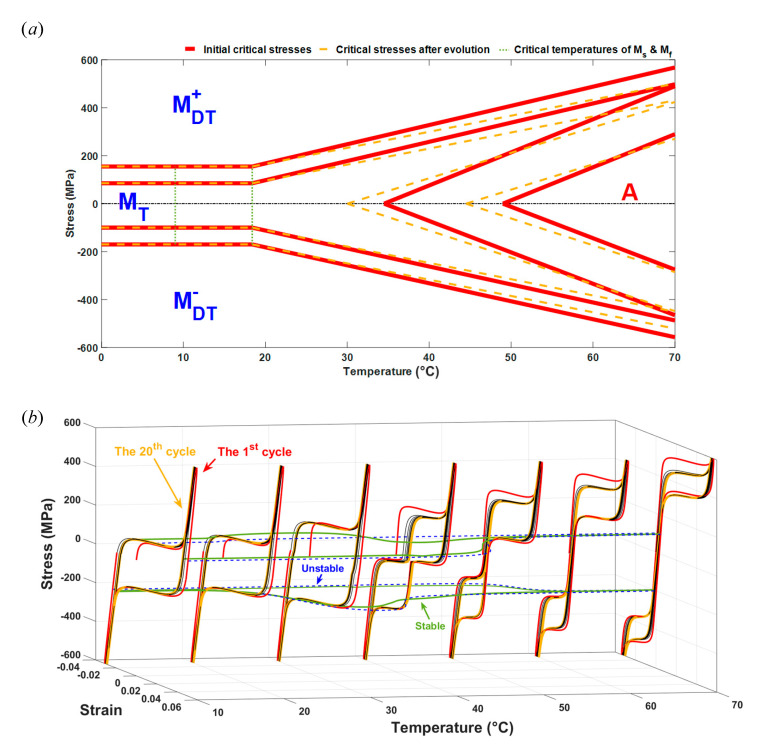
The temperature-dependent constitutive behavior of SMAs in the temperature range from 0 °C to 70 °C. (**a**) The asymmetric temperature-stress phase diagram predicting the critical stresses, red solid lines represent the initial critical stresses and the dark yellow dotted lines represent the critical stresses after evolution; (**b**) The strain–stress-temperature relationships of SMAs at various temperatures, red lines and the dark yellow lines represent the strain–stress relationships for the first and 20th cycles, respectively, the blue dashed lines represent the unstable strain-temperature relationship for the first cycle, and the green solid lines represent the stable strain-temperature relationship for the 20th cycle.

We use the applied stress, the range of which is 0 ± 600 MPa, in the following study and show the temperature-dependent constitutive behavior of SMAs in the temperature range from 0 °C to 70 °C in Figure 6 by using the proposed model. Regarding Figure 6, we first provide the asymmetric temperature-stress phase diagram predicted by the proposed model in Figure 6a. Note that in Figure 6a, all of the critical stresses, including their variations along with the temperature changes, are reflected, among which the red solid lines represent the critical stresses existing in the initial unstable cycle and the dark yellow dotted lines represent those existing in the final stable cycle. Different variants in SMAs, involving austenite (marked with “A”), twinned martensite (marked with “$M_{T}$”), tensile detwinned martensite (marked with ”$M_{DT}^{+}$”) and compressive detwinned martensite (marked with ”$M_{DT}^{-}$”), are divided by these critical stresses and shown in Figure 6a. For temperatures below M_s_ = 18.4 °C, the critical stresses in the initial cycle and after evolution are almost the same, which is also reflected in the results in Section 3.2. When the temperature increases above M_s_ = 18.4 °C, most of the absolute values of the critical stresses are relatively greater at the beginning and decrease after evolution due to the ratcheting effect of SMAs. A portion of absolute values of the critical stresses become greater after evolution, since $A_{s}$ and $A_{f}$ decrease during cycling [55]. Moreover, the phase diagram is not symmetrical for σ=0, which is due to the tensile-compressive asymmetry of SMAs. With the evolution rule of critical stresses reflected in Figure 6a, we plot the strain–stress-temperature relationships of SMAs for temperatures ranging from 0 °C to 70 °C in Figure 6b. It can be recognized in Figure 6b that the SMAs show an obvious shape-memory effect when T < 40 °C and pseudo-elasticity when T > 40 °C. When the temperature is equal to 40 °C, some transformation strains remain after the first tension-unloading process because the inverse martensitic transformation has not yet been completed by the time the unloading process has finished, and these strains are regarded as the initial strain for the following cycles. Regarding Figure 6b, we also find that the variation in the critical stress follows the results shown in the phase diagram in Figure 6a, which means that it increases with temperature increasing for both the unstable and stable cycles. Additionally, the area of the cyclic loops varies when the temperature changes and affects the energy dissipation of SMAs. Moreover, the strain-temperature relationships of SMAs can be seen in Figure 6b when the stress is fixed at 100 MPa and −200 MPa, respectively. We can conclude from these relationships that the strain variation range decreases when the cycle transits from the initial state to the stable state, especially for the higher temperature range. Some strain-temperature relationships show two strain bursts (regions where strain significantly varies with temperature). One burst is due to the evolution of the critical transition stresses and the other one is due to the feature transition of SMAs in the temperature range of $T\in [A_{s},A_{f}]$. The results shown in Figure 6 give us an idea of how to predict the tensile-compressive ratcheting deformations of SMAs in a changeable temperature environment.

**Figure 7:**
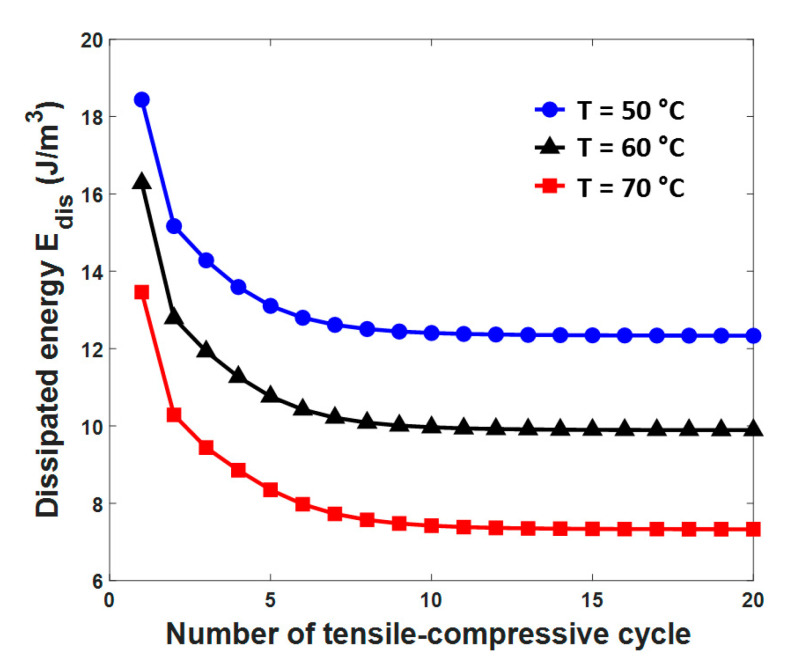
Variations in the dissipated energy $E_{dis}$ during tensile-compressive cycling under different temperatures, i.e., T = 50 °C, T = 60 °C, and T = 70 °C.

As we can see from Figure 6, the pseudo-elasticity can be clearly observed when T > 40 °C and the pseudo-elastic SMAs show excellent damping performance [57]; therefore, the energy dissipation of SMAs in that temperature range should be studied. The temperature-dependent dissipated energies of SMAs, $E_{dis}$, at three different temperatures, namely T = 50 °C, T = 60 °C, and T = 70 °C, are shown in Figure 7. From Figure 7, in which the loading with a stress range of 0±600 MPa is applied, we find that the dissipated energies of SMAs at these three temperatures all rapidly decline in the first ten cycles; then, the decreasing rate of these curves slow down; and finally, these dissipated energies tend towards a fixed value. Moreover, it can be clearly recognized that the SMAs dissipate more energy and show better damping performance in a lower-temperature environment, which is determined by the larger area of the hysteresis loops according to Figure 6b.

**Figure 8:**
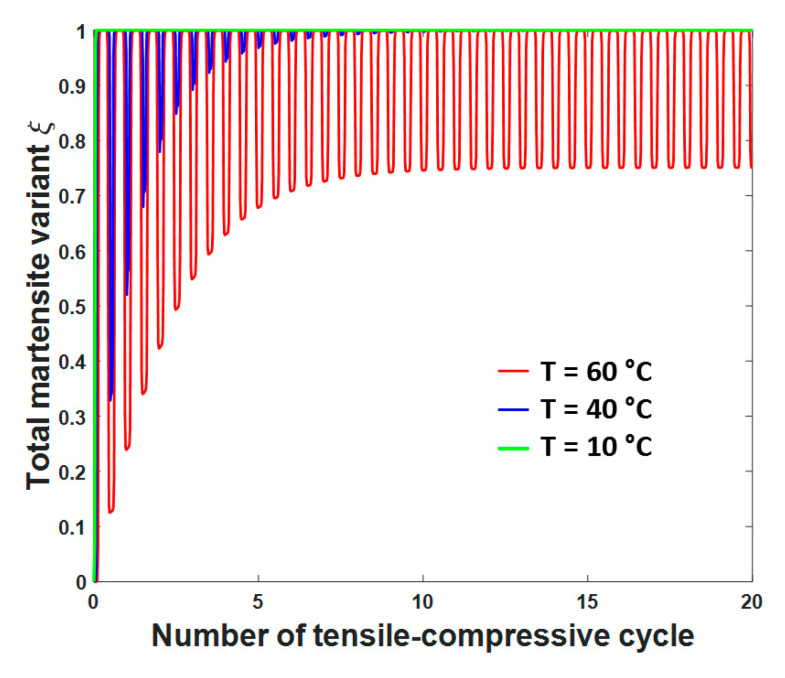
Variations in the total martensite variants ξ during tensile-compressive cycling under different temperatures, i.e., T = 10 °C, T = 40 °C, and T = 60 °C.

According to the proposed model, we show the variation in total martensite variants ξ in SMAs during cycling at different temperatures, as shown in Figure 8. Regarding Figure 8, the variations in ξ at T = 10 °C, T = 40 °C, and T = 60 °C, are described, the peak values of which all approach 1 when the applied stress under tension or compression reaches its peak value. Since obvious pseudo-elasticity can be observed when T = 60 °C, it can be seen from Figure 8 that the phase transformation between austenite and martensite is still in progress, with the value of ξ continuously fluctuating at that temperature. When the temperature decreases to 40 °C, the fluctuation of ξ becomes weaker after approximately seven tensile-compressive cycles, mainly due to the irreversible martensite and partial irreversible strain induced by the shape-memory effect of SMAs. When an even lower temperature is reached, i.e., T = 10 °C, the shape-memory effect becomes the main characteristic of SMAs; thus, the value of ξ reaches one at the end of the first tension or compression process and remains at one in the following cycles, as no phase transformation occurs afterwards.

**Figure 9:**
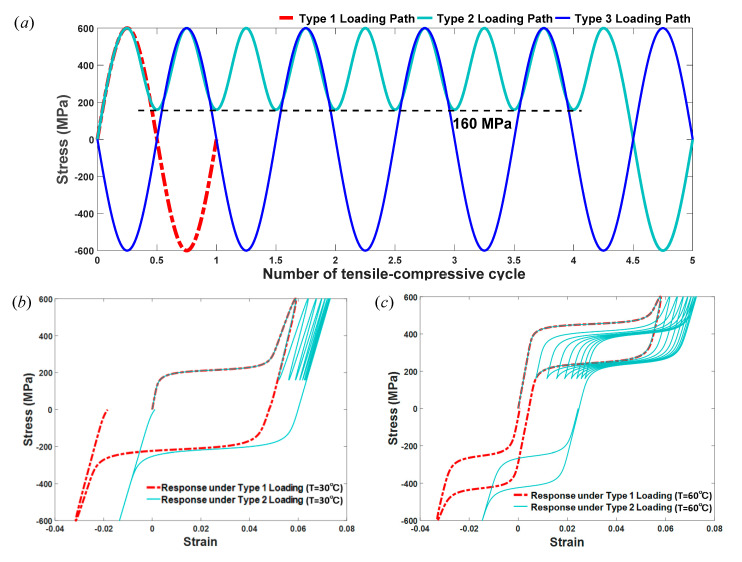

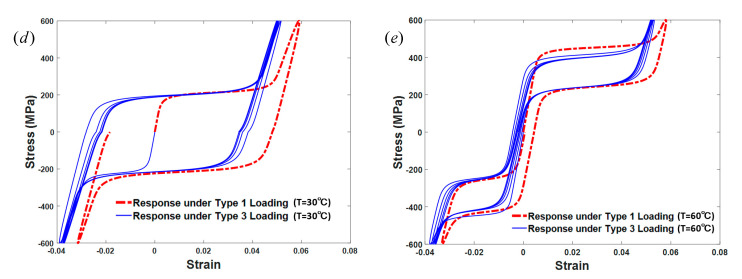
The temperature-dependent constitutive behavior of SMAs under three different types of loading path. (**a**) The loading paths along with the increase in the tensile-compressive cycle; (**b**) The strain–stress relationship of SMAs under Type 2 loading at T = 30 °C; (**c**) The strain–stress relationship of SMAs under Type 2 loading at T = 60 °C; (**d**) The strain–stress relationship of SMAs under Type 3 loading at T = 30 °C; (**e**) The strain–stress relationship of SMAs under Type 3 loading at T = 60 °C.

Finally, we consider the mechanical behaviors of SMAs under the other two different loading paths, involving a complete cycle with several tensile reloadings, whose stress range is 380±220 MPa (Type 2 loading path), and cycles that begin with compression (Type 3 loading path), as shown in Figure 9a. Note that in Figure 9a, Type 1 loading path of a simple complete cycle in red dash dotted line is used as a reference for comparison. The strain–stress responses of SMAs under Type 2 loading at different temperatures, T = 30 °C and T = 60 °C, are shown in Figure 9b,c. Regarding Figure 9b, we note that the transformation strain and ratcheting strain exist before the first reloading process begins, which is due to the shape-memory effect of SMAs at T = 30 °C. When the reloading–unloading processes take place and are repeated several times, several approximately linear strain–stress relationships with a ratcheting strain that tends to saturate can be found. These processes finally affect the first compression-unloading process in Figure 9b, the residual strain of which after compressive unloading is smaller than that of a simple complete tensile-compressive cycle (Type 1 loading). At T = 60 °C, several complete subloops can be clearly observed under tension in Figure 9c due to the pseudo-elasticity of SMAs, and a smaller hysteresis loop with smaller ratcheting strain can be captured in the compressive direction, since the ratcheting deformation is almost saturated. In addition, the strain–stress responses of SMAs under Type 3 loading at temperatures of 30 °C and 60 °C are plotted in Figure 9d,e. According to Figure 9d,e, the shape-memory effect and pseudo-elasticity of SMAs can be clearly reflected when Type 3 loading is applied. However, comparing with the responses under Type 1 loading, we obtain that the peak strains under compression are always greater and those under tension are smaller when Type 3 loading is applied.

## 4. Concluding Remarks

This work mainly addresses the description of the thermomechanical behavior of SMAs involving tensile-compression asymmetry and the ratcheting effect by proposing a temperature-dependent constitutive model. By considering the cyclic accumulation of residual martensite and redefining the internal variables in SMAs, the proposed model can reasonably predict the temperature-dependent characteristics of SMAs, involving the pseudo-elasticity and the shape-memory effect, and describe the mechanical behavior at an arbitrary temperature under different loading cases. Comparisons between the predictions of the proposed constitutive model of SMAs and the corresponding experimental results show that the proposed model effectively predicts the main features of tensile-compression asymmetry and the ratcheting effect observed from the experimental findings.

## Figures and Tables

**Table 1:** Evolution of the accumulated correction functions $\varepsilon_{e}$ and ϑ for multiple cycles.

Cycle Number	Correction Function $\varepsilon_{e}$ Related to Phase Transition	Correction Function ϑ Related to Tensile-Compressive Conversion
Tension-unloading of the 1st cycle (1^+^)	0	0
Compression-unloading of the 1st cycle (1^−^)	$\varepsilon_{L}^{+}\xi_{\sigma \to 0}^{1^{+}}+\varepsilon_{e}^{1^{+}}\left(1-\xi_{\sigma \to 0}^{1^{+}}\right)$	$2\times \left(\varepsilon_{0}^{1^{-}}-\varepsilon_{e}^{1^{-}}-\vartheta^{1+}/2\right)$
Tension-unloading of the 2nd cycle (2^+^)	$\varepsilon_{L}^{-}\xi_{\sigma \to 0}^{1^{-}}+\varepsilon_{e}^{1^{-}}\left(1-\xi_{\sigma \to 0}^{1^{-}}\right)$	$2\times \left(\varepsilon_{0}^{2^{+}}-\varepsilon_{e}^{2^{+}}-\vartheta^{1^{-}}/2\right)$
Compression-unloading of the 2nd cycle (2^−^)	$\varepsilon_{L}^{+}\xi_{\sigma \to 0}^{2^{+}}+\varepsilon_{e}^{2^{+}}\left(1-\xi_{\sigma \to 0}^{2^{+}}\right)$	$2\times \left(\varepsilon_{0}^{2^{-}}-\varepsilon_{e}^{2^{-}}-\vartheta^{2^{+}}/2\right)$
Tension-unloading of the 3rd cycle (3^+^)	$\varepsilon_{L}^{-}\xi_{\sigma \to 0}^{2^{-}}+\varepsilon_{e}^{2^{-}}\left(1-\xi_{\sigma \to 0}^{2^{-}}\right)$	$2\times \left(\varepsilon_{0}^{3^{+}}-\varepsilon_{e}^{3^{+}}-\vartheta^{2^{-}}/2\right)$
Compression-unloading of the 3rd cycle (3^−^)	$\varepsilon_{L}^{+}\xi_{\sigma \to 0}^{3^{+}}+\varepsilon_{e}^{3^{+}}\left(1-\xi_{\sigma \to 0}^{3^{+}}\right)$	$2\times \left(\varepsilon_{0}^{3^{-}}-\varepsilon_{e}^{3^{-}}-\vartheta^{3^{+}}/2\right)$
……	……	……
Tension-unloading of the ith cycle (i^+^)	$\varepsilon_{L}^{-}\xi_{\sigma \to 0}^{{\left(i-1\right)}^{-}}+\varepsilon_{e}^{{\left(i-1\right)}^{-}}\left(1-\xi_{\sigma \to 0}^{{\left(i-1\right)}^{-}}\right)$	$2\times \left(\varepsilon_{0}^{i^{+}}-\varepsilon_{e}^{i^{+}}-\vartheta^{{\left(i-1\right)}^{-}}/2\right)$
Compression-unloading of the ith cycle (i^−^)	$\varepsilon_{L}^{+}\xi_{\sigma \to 0}^{i^{+}}+\varepsilon_{e}^{i^{+}}\left(1-\xi_{\sigma \to 0}^{i^{+}}\right)$	$2\times \left(\varepsilon_{0}^{i^{-}}-\varepsilon_{e}^{i^{-}}-\vartheta^{i^{+}}/2\right)$

**Note:** The superscript “$i^{\pm }$” represents the number of half-cycles; the subscript ”σ→0” represents that the stress becomes zero from tensile or compressive unloadings; and $\varepsilon_{0}^{i\pm }$ is the initial strain for the corresponding i± cycles.

**Table 2:** Material parameters used in the proposed model for pseudo-elastic SMAs at room temperature. (NiTi alloys - Ni, 50.2 at.%).

**Parameters Remaining Constant During the Cyclic Transformation**
$D_{M}^{DT(+)}=D_{M}^{DT(-)}=45\;GPa$, $D_{A}=72\;GPa$, $\varepsilon_{sa}^{+}/\varepsilon_{sa}^{-}=0.012/0.042$, $\xi_{ir\_\max}^{+}/\xi_{ir\_\max}^{-}=0.43/0.321$
**Initial and Stabilized Values of the Evolution Parameters**
$\sigma_{10}^{+}/\sigma_{11}^{+}=320\;MPa/175\;MPa$, $\sigma_{20}^{+}/\sigma_{21}^{+}=578\;MPa/433\;MPa$, $\sigma_{30}^{+}/\sigma_{31}^{+}=405\;MPa/317\;MPa$, $\sigma_{40}^{+}/\sigma_{41}^{+}=140\;MPa/60\;MPa$, $\sigma_{10}^{-}/\sigma_{11}^{-}=-481\;MPa/-461\;MPa$, $\sigma_{20}^{-}/\sigma_{21}^{-}=-650\;MPa/-630\;MPa$, $\sigma_{30}^{-}/\sigma_{31}^{-}=-480\;MPa/-400\;MPa$, $\sigma_{40}^{-}/\sigma_{41}^{-}=-330\;MPa/-273\;MPa$, $\varepsilon_{L0}^{+}/\varepsilon_{L1}^{+}=0.04/0.018$, $\varepsilon_{L0}^{-}/\varepsilon_{L1}^{-}=-0.015/-0.008$, $\kappa_{0}^{+}/\kappa_{1}^{+}=(1/20)/(1/60)$, $\kappa_{0}^{-}/\kappa_{1}^{-}=(1/40)/(1/60)$
**Parameters Governing the Evolution Rates of the Evolution Parameters**
$\gamma_{1}^{\pm }=\gamma_{2}^{\pm }=\gamma_{3}^{\pm }=\gamma_{4}^{\pm }=\gamma_{6}^{\pm }=0.001$, $\gamma_{5}^{\pm }=0.0001$, $\psi_{ir}=0.00015$, $\psi_{2}=0.0002$

**Table 3:** Material parameters used in the proposed model for shape-memory SMAs at room temperature. (NiTi alloys–Ni, 50.2 at.%).

**Parameters Remaining Constant During the Cyclic Transformation**
$D_{M}^{DT(+)}=D_{M}^{DT(-)}=45\;GPa$, $D_{A}=72\;GPa$, $\varepsilon_{sa}^{+}/\varepsilon_{sa}^{-}=0.015/0.005$, $\xi_{ir\_\max}^{+}/\xi_{ir\_\max}^{-}=0.43/0.321$
**Initial and Stabilized Values of the Evolution Parameters**
$\sigma_{10}^{+}/\sigma_{11}^{+}=103\;MPa/103\;MPa$, $\sigma_{20}^{+}/\sigma_{21}^{+}=343\;MPa/343\;MPa$, $\sigma_{10}^{-}/\sigma_{11}^{-}=-165\;MPa/-165\;MPa$, $\sigma_{20}^{-}/\sigma_{21}^{-}=-372\;MPa/-372\;MPa$, $\varepsilon_{L0}^{+}/\varepsilon_{L1}^{+}=0.065/0.062$, $\varepsilon_{L0}^{-}/\varepsilon_{L1}^{-}=-0.04/-0.032$, $\kappa_{0}^{+}/\kappa_{1}^{+}=(1/20)/(1/80)$ , $\kappa_{0}^{-}/\kappa_{1}^{-}=(1/20)/(1/80)$
**Parameters Governing the Evolution Rates of the Evolution Parameters**
$\gamma_{1}^{\pm }=\gamma_{2}^{\pm }=\gamma_{3}^{\pm }=\gamma_{4}^{\pm }=\gamma_{6}^{\pm }=0.001$, $\gamma_{5}^{\pm }=0.0001$, $\psi_{ir}=0.00015$, $\psi_{2}=0.0002$

**Table 4:** Material parameters used in the proposed model for SMAs at various temperatures. (NiTi-Cu alloys).

**Parameters Remaining Constant During the Cyclic Transformation**
$D_{M}^{DT(+)}=D_{M}^{DT(-)}=D_{M}^{T}=45\;GPa$, $D_{A}=72\;GPa$, $\varepsilon_{sa}^{+}/\varepsilon_{sa}^{-}=0.012/0.008$, $\xi_{ir\_\max}^{+}/\xi_{ir\_\max}^{-}=0.43/0.321$
**The material parameters governing the evolution of the critical stresses**
$\sigma_{s}^{cr+}/\sigma_{f}^{cr+}=85\;MPa/155\;MPa$, $\sigma_{s}^{cr-}/\sigma_{f}^{cr-}=-100\;MPa/-170\;MPa$, $C_{M0}^{+}/C_{M1}^{+}=8/6.7$, $C_{A0}^{+}/C_{A1}^{+}=13.8/10.6$, $C_{M0}^{-}/C_{M1}^{-}=-7.5/-6.8$, $C_{A0}^{-}/C_{A1}^{-}=-13.1/-11.2$, $M_{f}=9\;{}^{{}^{\circ}}C$, $M_{s}=18.4\;{}^{{}^{\circ}}C$, $A_{s}^{0}/A_{s}^{1}=34.5\;{}^{{}^{\circ}}C/30\;{}^{{}^{\circ}}C$, $A_{f}^{0}/A_{f}^{1}=49\;{}^{{}^{\circ}}C/44.5\;{}^{{}^{\circ}}C$
**Initial and Stabilized Values of Other Evolution Parameters**
$\varepsilon_{L0}^{+}/\varepsilon_{L1}^{+}=0.043/0.04$, $\varepsilon_{L0}^{-}/\varepsilon_{L1}^{-}=-0.023/-0.019$, $\kappa_{0}^{+}/\kappa_{1}^{+}=(1/20)/(1/30)$, $\kappa_{0}^{-}/\kappa_{1}^{-}=(1/20)/(1/30)$, $\kappa^{R}=1$
**Parameters Governing the Evolution Rates of the Evolution Parameters**
$\gamma_{1}^{\pm }=\gamma_{2}^{\pm }=\gamma_{3}^{\pm }=\gamma_{4}^{\pm }=\gamma_{6}^{\pm }=0.001$, $\gamma_{5}^{\pm }=0.0001$, $\psi_{ir}=0.00015$, $\psi_{2}=0.0002$
